# Ethnic differences in ischemic stroke subtypes in young-onset stroke: the Stroke Prevention in Young Adults Study

**DOI:** 10.1186/s12883-015-0461-7

**Published:** 2015-10-29

**Authors:** Megh M. Trivedi, Kathleen A. Ryan, John W. Cole

**Affiliations:** Department of Neurology, University of Maryland School of Medicine, Baltimore, MD USA; Veterans Affairs Medical Center, Baltimore, MD USA

**Keywords:** Stroke, Risk factors, Ethnicity, Young-onset

## Abstract

**Background:**

Prior studies indicate that young African-Americans (AA) have a greater frequency of ischemic stroke than similarly aged European-Americans (EA). We hypothesized that differences in stroke subtype frequency mediated through sex and differing risk factor profiles may play a role in ethnicity-specific stroke. Utilizing our biracial young-onset stroke population, we explored these relationships.

**Methods:**

Fifty nine hospitals in the Baltimore-Washington area participated in a population-based study of young-onset stroke in men (218-AA, 291-EA) and women (219-AA, 222-EA) aged 16–49. Data on age, sex, ethnicity and stroke risk factors (hypertension (HTN) and smoking) were gathered through standardized interview. A pair of vascular neurologists adjudicated each case to determine TOAST subtype. Logistic regression analyses evaluating for differences in stroke risk factors by TOAST subtype were performed.

**Results:**

Analyses controlling for age and sex demonstrated that AA were more likely to have a lacunar stroke than EA (OR = 1.61; 95 % CI = 1.12–2.32; *p* = 0.011) when utilizing the other TOAST subtypes as the reference group. This effect was mediated by HTN, which increases the risk of lacunar stroke (OR = 2.03; 95 % CI = 1.38–2.98; *p* = 0.0003) and large artery stroke (OR = 1.70; 95 % CI = 1.01–2.88; *p* = 0.048) when controlling for sex, ethnicity, and age. Cases below age 40 were more likely to have a cardioembolic stroke than those above age 40 (OR = 1.62; 95 % CI = 1.15–2.27; *p* = 0.006), controlling for sex and ethnicity. Lastly, current smokers were more likely to have a large artery stroke than non-smokers (OR = 1.79; 95 % CI = 1.08–2.98; *p* = 0.024).

**Conclusions:**

Our population-based data demonstrate ethnic differences in ischemic stroke subtypes. These findings may help clarify mechanisms of stroke in young adults which may in part be driven by ethnic-specific differences in early-onset traditional risk factors, thereby indicating differing emphasis on workup and prevention.

## Background

Many prior studies have demonstrated that African-Americans (AA) have a higher incidence of stroke, more severe strokes, and higher stroke mortality as compared to European-Americans (EA) [1]. Such ethnic disparities are thought to be driven by a higher prevalence or severity of stroke risk factors, lower socioeconomic status among AA, and due to biological differences between AA and EA. Further, age-of-onset ethnic disparities have been consistently demonstrated with age-adjusted stroke incidence rates higher in AA than in EA [2, 3]. One publication reported the relative excess in deaths from stroke among AA compared with EA was most manifest in the population aged <65 years, in which the AA/EA mortality ratio was 3.7 among men aged 45–54 years [3]. While these and other studies clearly demonstrate differences in age-related ethnic stroke incidence and mortality, few studies have included evaluations of stroke etiology or subtype, particularly in younger populations. Early-onset stroke, here defined as onset <50 years of age, accounts for approximately 10–15 % of all strokes [4, 5] and often causes affected individuals to endure a greater number of years of morbidity, lost years of productivity, and an increased long-term familial burden. Such facts prompt efforts to better understand young-onset stroke, the specific groups at risk, and identify factors that can be optimized earlier in life to minimize future risk.

Currently stroke is the fifth leading cause of death in the United States and the leading cause of disability [6]. Ischemic strokes constitute ~85 % of all strokes and are often classified by subtype using the Trial of Org 10172 in Acute Stroke Treatment, also known as TOAST [7]. Each TOAST subtype is primarily defined by differing etiologic mechanisms, which can be used to help define workup, prognosis, and follow-up needs; thereby making TOAST an important classification schema for all physicians that treat ischemic stroke. Beyond the differing stroke subtypes, we further emphasize that physicians should also be aware that ethnic differences in subtype rates exist and that risk factor profiles differ by subtype. Other non-modifiable factors such as age and sex also play key roles in stroke susceptibility. For example, the incidence of stroke is also known to be slightly higher in males than females [8] and the chance of having a stroke approximately doubles for each decade of life after age 55 [9]. Such non-modifiable risk factors (i.e. ethnicity, age, and sex) can be utilized to stratify at-risk populations into fixed groups, which can then be evaluated for differences in stroke subtypes and modifiable risk factors, such as hypertension and smoking among others. While several prominent papers have been published analyzing these relationships in older populations, there is a paucity of such research in young adults. We hypothesize that differences in stroke subtype frequencies are mediated through sex and differing risk factor profiles, thereby playing an important role in ethnicity-specific stroke among young adults.

## Methods

Case-only data from a biracial young-onset stroke population was utilized to explore the relationships between risk factors and stroke subtypes in young adults of differing ethnicity and sex. Cases aged 15–49 with a first ischemic stroke were identified by discharge surveillance from one of 59 hospitals in the greater Baltimore-Washington area and by direct referral from regional neurologists. Cases were recruited in 3 different time periods: Stroke Prevention in Young Women-1 (SPYW-1) conducted from 1992 to 1996, Stroke Prevention in Young Women-2 (SPYW-2) conducted from 2001 to 2003, and Stroke Prevention in Young Men (SPYM) conducted from 2003 to 2007. SPYW-1 included cases aged 15–44 years recruited within 1 year of stroke and SPYW-2 and SPYM included cases aged 15–49 recruited within 3 years of stroke. The methods for discharge surveillance and chart abstraction have been described previously [10, 11]. Data on age, sex and ethnicity were gathered through standardized interview. Risk factors including hypertension, diabetes mellitus, and myocardial infarction were determined by asking the study participant (or his/her proxy if the participant was unable to answer) if he/she had ever been told by a physician that he/she had the condition. Smoking status was defined as current (within the last month) versus former/never combined. Hyperlipidemia was not included since it was not a study measurement and self-reporting by participants was inadequate.

Our study population consisted of 1001 stroke patients with 51 patients excluded from the current study since they were neither AA nor EA. Of the 950 remaining patients, 509 were male (218-AA, 291-EA) and 441 were female (219-AA, 222-EA). A pair of vascular neurologists adjudicated each case to determine TOAST subtype. The TOAST system classifies ischemic strokes into five categories, including: (1) lacunar (small vessel), (2) large artery (atherosclerotic), (3) cardioembolic, (4) other determined etiology, and (5) undetermined etiology (cryptogenic). Based on the presenting clinical picture, a patient was assigned to one of these five categories with either probable or possible certainty. Under the TOAST criteria, those patients whose stroke may be a result of multiple possible etiologies are included in the category of undetermined etiology. A written informed consent for participation in the study was obtained from each participant or when appropriate from the participant’s guardian. This protocol was approved by University of Maryland, Baltimore Institutional Review Board prior to study commencement.

SAS version 9.2 (Cary, NC) was used to perform logistic regression to determine the odds of a group having a specific subtype of stroke over another group. Three pairs (of groups) were analyzed: female vs. male, EA vs. AA, and age of <40 (16–39) vs. >40 (40–50) years old. Forty was the mean age of our study population, providing the reasoning behind our age dichotomization. Modifiable risk factors were added to determine if they explained the significance of the results. If the inclusion of a variable significantly reduced the odds of a specific subtype of stroke for a specific group, then that variable was considered a predisposing factor for that subtype of stroke for that specific group. Two analyses utilizing differing reference groups were performed; 1) *Analysis 1* compared each individual TOAST subtype against all other subtypes combined; this analysis provides information regarding the odds of having a specific subtype of stroke as compared to any other subtype, and; 2) *Analysis 2* compared each individual TOAST subtype against the cryptogenic subtype; this analysis provides information regarding the odds of having a specific identifiable subtype of stroke as compared to a stroke of unknown etiology (i.e. cryptogenic). Of note, 71 cases classified as other determined etiologies by TOAST were excluded from these analyses and included the following etiologies: dissection (8-AA, 17-EA), hypercoagulability (11-AA, 13-EA), hypertensive encephalopathy (3-AA, 1-EA), autoimmune related (2-AA, 4-EA), and other rarer causes (*n* = 12). Also, one cardioembolic stroke case and two cryptogenic stroke cases were missing complete risk factor information precluding them from inclusion in the risk factor based analyses.

## Results

### Population characteristics

Prior to computing the odds ratios of each stroke subtype, the population characteristics were examined to look for any trends, as well as risk factor candidates for further analysis. Table 1 shows the population characteristics stratified by TOAST subtype, and Table 2 shows the population characteristics stratified across TOAST subtypes. In Table 1, a single stroke subtype is taken and percentages are calculated for each population group. For example, of all 142 people who suffered lacunar strokes, 60.56 % were males (86 males). This means that the remaining 39.44 % were females (56 females). Table 2 examines the population in a different manner by taking a subgroup and examining frequency of different stroke subtypes. For example, when comparing males and females, 16.90 % of males had a lacunar stroke compared to 12.70 % of females. This prompted us to further analyze whether men had greater odds of having a lacunar stroke than females, matched for ethnicity and age. In a similar fashion, the frequency of lacunar stroke was seen across different groups and different risk factors. Of the four risk factors (HTN, smoking, DM and MI), the risk factors of DM and MI were excluded because of low number of subjects (DM:155; MI:48) in the ethnically stratified subtype cells. Of note, the distribution of diabetes by subtype and ethnicity was as follows; CE = 21 (AA-16, EA-5), LAA = 13 (AA-4, EA-9), lacunar = 43 (AA-26, EA-17), cryptogenic = 75 (AA-49, EA-26). Ultimately, only HTN and smoking were included in the analyses.

**Table 1:** Population characteristics stratified by TOAST subtype

	Lacunar *N* = 142	Large artery *N* = 68	Cardioembolic *N* = 182	Other determined *N* = 71	Cryptogenic *N* = 487
Male (%)	60.56	66.18	57.69	25.35	52.36
AA (%)	56.34	39.71	45.60	38.03	45.17
Age ≥40 (%)	85.21	85.29	59.89	47.89	65.91
HTN (%)	61.27	55.88	39.01	21.13	37.73
Smoking (%)	46.48	57.35	35.36	40.85	45.38
DM (%)	30.28	19.12	11.54	4.23	15.43
MI (%)	4.23	4.41	8.24	2.82	4.54

Example: Of small vessel strokes, 60.56 % were males

**Table 2:** Population characteristics across TOAST subtypes

	Lacunar (%)	Large artery (%)	Cardioembolic (%)	Other determined (%)	Cryptogenic (%)	Total
Male	16.90	8.84	20.63	3.54	50.10	100 %
Female	12.70	5.22	17.46	12.02	52.61	100 %
AA	18.31	6.18	18.99	6.18	50.34	100 %
EA	12.09	7.99	19.30	8.58	52.05	100 %
Age ≥40	18.82	9.02	16.95	5.29	49.92	100 %
Age <40	6.84	3.26	23.78	12.05	54.07	100 %
HTN	22.08	9.64	18.02	3.81	46.45	100 %
No HTN	9.93	5.42	20.04	10.11	54.51	100 %
Smoking	15.75	9.31	15.27	6.92	52.74	100 %
No smoking	14.34	5.47	22.08	7.92	50.19	100 %
DM	27.74	8.39	13.55	1.94	48.39	100 %
No DM	12.47	6.93	20.28	8.56	51.76	100 %
MI	12.50	6.25	31.25	4.17	45.83	100 %
No MI	15.11	7.22	18.56	7.67	51.44	100 %

Example: Of all males with ischemic stroke, 16.90 % had small vessel stroke, 8.84 % had large artery stroke, 20.63 % had cardioembolic stroke, etc

#### Analysis 1: individual TOAST subtypes compared against all other subtypes combined

The results of *Analysis 1* are seen in Tables 3 and 4. Table 3 shows the basic model of the logistic regression analysis. The model calculates the odds of having a subtype of stroke for one subgroup while controlling for the other subgroups. Table 4 shows the odds once smoking and HTN are added into the model. If a statistically significant result in Table 3 is no longer statistically significant in Table 4, we interpreted this to indicate that either smoking or HTN are mediating the increase in odds risk. Further analyses were then conducted to determine which risk factor mediated the risk. No statistically significant sex differences were noted in stroke subtype risk. AA were more likely to have a lacunar stroke than EA (OR = 1.61; 95 % CI = 1.12–2.32; *p* = 0.011), controlling for sex and age. This effect may be mediated by HTN, which increases the risk of lacunar stroke (OR = 2.03; 95 % CI = 1.38–2.98; *p* = 0.0003) and large artery stroke (OR = 1.70; 95 % CI = 1.01–2.88; *p* = 0.048) when controlling for sex, ethnicity, and age. Patients above age 40 were more likely to have lacunar stroke (OR = 2.97; 95 % CI = 1.82–4.86; *p* <0.0001) and large artery stroke (OR = 2.77; 95 % CI = 1.39–5.55; *p* = 0.004), controlling for sex and ethnicity. Conversely, patients below age 40 were more likely to have a cardioembolic stroke (OR = 1.62; 95 % CI = 1.15–2.27; *p* = 0.006), controlling for sex and ethnicity. Lastly, current smokers were more likely to have a large artery stroke than non-smokers (OR = 1.79; 95 % CI = 1.08–2.98; *p* = 0.024).

**Table 3:** *Analysis 1*: TOAST subtype vs. all other subtypes combined

	Small vessel (*n* = 142)	Large artery (*n* = 68)	Cardioembolic (*n* = 182)	Cryptogenic (*n* = 487)
Sex (1 = male)	1.26 *p* = 0.226	1.50 *p* = 0.132	1.34 *p* = 0.086	0.92 *p* = 0.544
	[0.87, 1.83]	[0.89, 2.55]	[0.96, 1.88]	[0.71, 1.20]
Ethnicity (1 = AA)	**1.61** ***p*** **= 0.011**	0.75 *p* = 0.271	1.02 *p* = 0.895	0.94 *p* = 0.610
	**[1.12, 2.32]**	[0.45, 1.25]	[0.74, 1.42]	[0.72, 1.21]
Age (≥40)	**2.97** ***p*** **<0.0001**	**2.77** ***p*** **= 0.004**	**0.62** ***p*** **= 0.006**	0.86 *p* = 0.298
	**[1.82, 4.86]**	**[1.39, 5.55]**	**[0.44, 0.87]**	[0.65, 1.14]

Logistic regression performed with the three groups of sex, ethnicity, and age - comparing stroke subtype risk against all other stroke subtypes combined. The results for sex and controlled by ethnicity and age. The results for ethnicity are controlled by sex and age. The results for age are controlled by sex and ethnicity. Significant results are bolded

**Table 4:** *Analysis 1*: TOAST subtype vs. all other subtypes combined with smoking and HTN included in the regression model

	Small vessel (*n* = 142)	Large artery (*n* = 68)	Cardioembolic (*n* = 181)	Cryptogenic (*n* = 485)
Sex	1.17 *p* = 0.42	1.50 *p* = 0.140	1.32 *p* = 0.115	0.96 *p* = 0.742
(1 = male)	[0.80, 1.71]	[0.88, 2.56]	[0.94, 1.85]	[0.74, 1.25]
Ethnicity	1.36 *p* = 0.109	0.64 *p* = 0.092	1.03 *p* = 0.845	1.01 *p* = 0.950
(1 = AA)	[0.93, 1.99]	[0.38, 1.08]	[0.74, 1.45]	[0.77, 1.31]
Age	**2.56** ***p*** **= 0.0002**	**2.38** ***p*** **= 0.016**	**0.65** ***p*** **= 0.014**	0.91 *p* = 0.530
(≥40)	**[1.56, 4.22]**	**[1.18, 4.83]**	**[0.46, 0.92]**	[0.69, 1.21]
Current smoker	1.05 *p* = 0.804	**1.79** ***p*** **= 0.024**	**0.66** ***p*** **= 0.016**	1.12 *p* = 0.380
(1 = yes)	[0.73, 1.52]	**[1.08, 2.98]**	**[0.47, 0.93]**	[0.87, 1.46]
HTN	**2.03** ***p*** **= 0.0003**	**1.70** ***p*** **= 0.048**	0.94 *p* = 0.713	**0.74** ***p*** **= 0.027**
(1 = yes)	**[1.38, 2.98]**	**[1.01, 2.88]**	[0.66, 1.33]	**[0.56, 0.97]**

Logistic regression performed with the three groups of sex, ethnicity, and age - comparing stroke subtype risk against all other stroke subtypes combined. The risk factors of smoking and HTN were added into the basic model. Significant results are bolded

#### Analysis 2: individual TOAST subtypes compared against cryptogenic subtype

The results of *Analysis 2* are seen in Tables 5 and 6. This analysis differs from A*nalysis 1* in that it evaluates the odds of having a stroke of an identifiable subtype rather than a cryptogenic stroke. Again, no statistically significant sex differences were noted in stroke subtype risk. Compared to cryptogenic stroke, AA are more likely to have a lacunar stroke than EA (OR = 1.57; 95 % CI = 1.06–2.31; *p* = 0.02), controlling for sex and age. This effect may be mediated by HTN, which increases the risk of lacunar stroke (OR = 2.18; 95 % CI = 1.46–3.25; *p* = 0.0001) and large artery stroke (OR = 1.88; 95 % CI = 1.10–3.21; *p* = 0.02) against cryptogenic stroke, when controlling for sex, ethnicity, and age. Compared to cryptogenic stroke, patients above age 40 were more likely to have lacunar stroke (OR = 2.78; 95 % CI = 1.68–4.61; *p* <0.0001) and large artery stroke (OR = 2.78; 95 % CI = 1.37–5.63; *p* = 0.005), controlling for sex and ethnicity. Unlike *Analysis 1*, this analysis did not demonstrate that patients below age 40 were more at risk of cardioembolic stroke, although the trend persisted. Furthermore, current smokers were less likely to have a cardioembolic stroke against cryptogenic stroke than non-smokers (OR = 0.67; 95 % CI = 0.47–0.95; *p* = 0.03). Conversely, they are more likely to have a cryptogenic stroke.

**Table 5:** *Analysis 2*: TOAST subtype vs. Cryptogenic stroke

	Small vessel (*n* = 142)	Large artery (*n* = 68)	Cardioembolic (*n* = 182)
Sex	1.35 *p* = 0.14	1.50 *p* = 0.15	1.33 *p* = 0.12
(1 = male)	[0.91, 2.00]	[0.87, 2.59]	[0.93, 1.89]
Ethnicity	**1.57** ***p*** **= 0.02**	0.82 *p* = 0.47	1.06 *p* = 0.74
(1 = AA)	**[1.06, 2.31]**	[0.49, 1.39]	[0.75, 1.50]
Age	**2.78** ***p*** **<0.0001**	**2.78** ***p*** **= 0.005**	0.73 *p* = 0.09
(≥40)	**[1.68, 4.61]**	**[1.37, 5.63]**	[0.51, 1.04]

Logistic regression performed with the three groups of sex, ethnicity, and age - comparing stroke subtype risk against cryptogenic stroke. The results for sex are controlled by ethnicity and age. The results for ethnicity are controlled by gender and age. The results for age are controlled by sex and ethnicity. Significant results are bolded

**Table 6:** *Analysis 2*: TOAST Subtype vs. Cryptogenic Stroke with smoking and HTN included in the regression model

	Small vessel (*n* = 142)	Large artery (*n* = 68)	Cardioembolic (*n* = 181)
Sex	1.30 *p* = 0.21	1.50 *p* = 0.15	1.29 *p* = 0.17
(1 = male)	[0.87, 1.94]	[0.86, 2.62]	[0.90, 1.84]
Ethnicity	1.34 *p* = 0.15	0.70 *p* = 0.20	1.04 *p* = 0.84
(1 = AA)	[0.90, 2.00]	[0.41, 1.21]	[0.72, 1.49]
Age	**2.42** ***p*** **= 0.0007**	**2.36** ***p*** **= 0.02**	0.72 *p* = 0.08
(≥40)	**[1.45, 4.05]**	**[1.15, 4.84]**	[0.50, 1.04]
Current smoker	0.97 *p* = 0.89	1.63 *p* = 0.07	**0.67** ***p*** **= 0.03**
(1 = yes)	[0.66, 1.44]	[0.96, 2.75]	**[0.47, 0.95]**
HTN	**2.18** ***p*** **= 0.0001**	**1.88** ***p*** **= 0.02**	1.12 *p* = 0.56
(1 = yes)	**[1.46, 3.25]**	**[1.10, 3.21]**	[0.77, 1.63]

Logistic regression performed with the three groups of sex, ethnicity, and age - comparing stroke subtype risk against cryptogenic stroke. Logistic regression performed with the three groups of sex, ethnicity, and age. The risk factors of smoking and HTN were added into the basic model. Significant results are bolded

## Discussion

Our data demonstrates that young AAs are more likely to experience a lacunar stroke than young EAs, as explained by an increased incidence of HTN. Younger-young individuals, those less than 40 years old, are more likely to experience a cardioembolic stroke, while individuals in the 40–50 year old age group are more likely to experience a lacunar or large vessel stroke. In general, current smokers are more likely to experience a large artery stroke, while individuals with HTN are more likely to experience a lacunar or large artery stroke. Of note, our two analyses which utilize differing reference groups produced similar results less that *Analysis 2* (cryptogenic stroke as reference) did not demonstrate that patients below age 40 were more at risk of cardioembolic stroke; this may relate to a decreased reference sample size.

Our results add to the growing literature demonstrating ethnic differences in stroke subtype proportions [1, 12, 13], further inferring on these relationships in a younger-onset population. In 2012 Song et al. retrospectively evaluated 350 acute ischemic stroke cases (mean age of 63) on the basis of TOAST classification. In contrast to our findings, their older population demonstrated similar proportions of lacunar strokes in the AA and EA cohorts. As consistent with our findings, similar proportions of cardioembolic stroke were reported in their AA and EA cohorts. In another study [14], a cohort of 511 patients between 18 and 49 years of age (mean age of 39.8) demonstrated no significant sex-based differences in the proportion of small- and large-vessel disease, and stroke of undetermined etiology, although cardioembolism (and substance abuse) predominated in men as compared with women. In contrast, and limiting potential comparisons to our study, 44 % of the young stroke patients (and almost 60 % of the women) had nontraditional etiologies for stroke (i.e. prothrombotic states, migraine-related conditions, substance abuse, cervical artery dissection, cerebral venous thrombosis, inflammatory and miscellaneous vasculopathies, and pathological conditions related to pregnancy, postpartum, fibromuscular dysplasia or Moyamoya syndrome) [14]. Although sex differences have also been demonstrated in other studies with men experiencing more strokes than women [15, 16], the precise mechanisms for these differences remains uncertain. In our study, among the lacunar, large-artery and cardioembolic subtypes we did not find any significant sex differences in our young-onset population, although by percentage, females were more likely to have a stroke of other determined etiology.

Other prior studies have demonstrated differences in stroke incidence between ethnic groups at young ages. In the Northern Manhattan Stroke Study young AA aged 20–44 were found to be 2.4 times more likely to have a stroke than similarly aged EA [17]. Other more recent studies have demonstrated that the incidence of stroke in the young is on the rise. For example, an analysis of temporal trends of stroke in the Greater Cincinnati/Northern Kentucky region demonstrated that the incidence of ischemic stroke in adults below age 55 has risen from 12.9 % in 1993/1994 to 18.6 % in 2005 [18]. In our data, when controlling for sex and ethnicity, we found that younger adults below age 40 were 1.62 times more likely to experience cardioembolic stroke than adults between ages 40–50, whereas adults in the 40–50 age group were 2.97 and 2.77 times more likely to experience lacunar and large artery strokes, respectively.

While ethnicity, age, and sex are major non-modifiable risk factors for stroke, the modifiable risk factors remain a critical intervention point for physicians working to reduce future stroke risk. Smoking and hypertension have long been associated with a higher risk of stroke. Smoking increases stroke risk through multiple mechanisms [19] and does so in a dose-dependent type fashion [20]. Ethnic differences in stroke risk as associated with smoking remain uncertain. In contrast, evidence exists demonstrating a differing ethnic response to hypertension as associated with stroke risk, with a greater risk among AA as compared to EA [21]. Many of these traditional risk factors develop during young-adulthood. One study examined the risk factor profiles of ischemic stroke under the ASCO classification [22] in patients age 16–54 and found that smoking, diabetes, hypertension and low HDL-cholesterol are significant risk factors for atherothrombosis, and that hypertension is a significant risk factor for small vessel disease in young adults [23]. While few studies exist that comprehensively evaluated ethnic and risk factors differences in young-onset stroke, some studies have evaluated such strata individually. For example, a Greek study evaluating the incidence of ischemic stroke in young adults aged 15–45 demonstrated that smoking was the most frequent risk factor, with 59.3 % of the young strokes actively smoking [24]. In our young-onset stroke study, we found that HTN increases the risk of lacunar stroke by 2.03 times and large artery stroke by 1.71 times, when controlling for sex, ethnicity, and age. Furthermore, our data show that the increased risk for lacunar stroke in AA compared to EA is mediated by HTN. Despite the fact that we did not find smoking to mediate the ethnic differences in stroke occurrence, we did find that current smokers were 1.81 times more likely to have a large artery stroke than non-smokers.

Another important point to consider is that a significant proportion of the patients in our study had cryptogenic stroke (51.3 %). While some prior studies have shown similar percentages of cryptogenic stroke [11, 12], this is somewhat higher than other studies evaluating young-onset ischemic stroke [24, 25]. This distribution not only reduced the sample sizes of the other subtypes, the heterogeneity of this group limited our ability to determine which risk factors were primarily responsible for the stroke.

Our study has several limitations. One primary limitation was the small sample sizes in several of the ethnically-stratified TOAST-subtype cells, thereby precluding investigations of several common traditional risk factors (i.e. DM, prior MI). Furthermore, the effects of dyslipidemia could not be evaluated secondary to inconsistent lipid data collection. Additionally, our study only includes those of EA and AA descent; other ethnicities present in the US population (e.g. Hispanics, Asians) could not be explored. Again, as based on limitations of ethnic- and subtype-specific sample sizes we were unable to analyze other stroke etiologies (dissection, hematological, etc.) and other risk factors, including drug abuse, which are commonly seen in younger stroke populations. However, we did not exclude such cases, which may also influence our findings. For example, 8 cases with acute cocaine use (within 24 h) were included in our analyses; their TOAST subtypes were: Cardioembolic *n* = 1, Lacunar *n* = 1, Other Determined *n* = 1 and Cryptogenic *n* = 5. Our study also only evaluated a single US geographic region, specifically the areas including and surrounding the Baltimore/Washington-DC metro region limiting the application of our results to other populations; within the US the distribution of traditional and non-traditional risk factors can differ vastly by geographical region. As such, similar studies conducted across the US and world-wide are needed to verify our findings. Also as mentioned previously, TOAST classifies strokes to one of five categories with either probable or possible certainty, with patients whose strokes that may have resulted via several possible etiologies being included in the category of undetermined etiology. Perhaps in the future improved subtyping methodologies such as the Causative Classification Algorithm (https://ccs.mgh.harvard.edu/ccs_title.php) [26–28] will allow patients to be better categorized into distinct stroke subtypes, thereby allowing for more refined classifications and analyses.

While our study was able to identify several groups predisposed to specific stroke subtypes and the risk factors driving these relationships, additional studies are needed to further identify other high risk ethnic-specific groups and refine risk estimates in other non-traditional risk factors, such as migraine headache and evolving risk factors such as e-cigarettes.

## Conclusions

Our population-based case-only analyses demonstrate ethnic differences in ischemic stroke subtypes among young adults. These findings may help clarify mechanisms of stroke in young adults, which may be partially driven by ethnic differences in the onset of early traditional stroke risk factors. Awareness of these associations may improve a physician’s ability to recognize predisposing risk factors for a specific subtype of ischemic stroke within an individual, thereby indicating a differing emphasis on primary and secondary stroke prevention and infer on the workup after a stroke has occurred.
